# Validity of a Self-administered Food Frequency Questionnaire Used in the 5-year Follow-up Survey of the JPHC Study Cohort I to Assess Dietary Fiber Intake: Comparison with Dietary Records

**DOI:** 10.2188/jea.13.1sup_106

**Published:** 2007-11-30

**Authors:** Satoshi Sasak, Yasuhiro Matsumura, Junko Ishihara, Shoichiro Tsugane

**Affiliations:** 1 Epidemiology and Biostatistics Division, National Cancer Center Research Institute East.; 2 National Institute of Health and Nutrition.; 3 Cancer Information and Epidemiology Division, National Cancer Center Research Institute.

**Keywords:** validity, questionnaire, dietary fiber, diet

## Abstract

We examined the validity of a food frequency questionnaire (FFQ) used in the 5-year follow-up survey of the JPHC study for estimation of dietary fiber intake by comparing the intake estimated with dietary records (DR). We developed a food composition table for dietary fiber for the food items included in the FFQ using a substitution method. The Spearman correlation coefficients were slightly higher in crude values (0.48-0.51 in men, 0.40-0.45 in women) than in energy-adjusted values (0.43-0.44 in men, 0.36-0.40 in women). The correlation coefficients of food group-specific dietary fiber (crude) were 0.26 and 0.27 for vegetables, and 0.62 and 0.49 for fruits in men and women, respectively. The mean intakes assessed with the FFQ and DR were not statistically different either for water-soluble or -insoluble fiber in both men and women. However, the fruit fiber assessed with FFQ was significantly overand the vegetable fiber was underestimated compared with those assessed with DRs. The FFQ can be used for ranking individuals for dietary fiber intakes in epidemiologic studies, despite the difficulty in estimating mean intake.

Several epidemiologic studies have reported a possible association of dietary fiber with cancer, cardiovascular disease and diabetes mellitus.^1^^-^^3^ However, because of the lack of a reliable food composition table of dietary fiber for Japanese foods,^4^ nutritional epidemiologic studies on dietary fiber intake and health have been hampered in Japanese populations. Some previous studies developed a dietary fiber food composition table for their specific use,^5^^,^^6^ but they were not available for use in other studies.

We therefore developed a dietary fiber food composition table specifically for a self-administered semiquantitative food frequency questionnaire (FFQ) used in the 5-year follow-up survey of the JPHC study using a substitution method.^7^ Then the validity of the FFQ was examined by comparison with estimated intakes assessed with dietary records (DR).

## MATERIALS AND METHODS

### Development of Food Composition Table

The Standard Tables of Food Composition in Japan, 4th revised edition by Science and Technology Agency^4^ listed 227 foods with dietary fiber composition, and the 5th revised edition added 102 foods.^8^ Based on these 329 foods, we substituted the composition of dietary fiber for the other 127 foods using two other food composition databases on dietary fiber^9^^,^^10^ according to a substitution method developed by Sasaki et al.^7^ To make the development procedure easier and more practical, we limited the foods to those that appeared in the DR used for the validation study of FFQ.

### Validation of FFQ

Dietary fiber intake was calculated from DR and FFQ by dividing into water-soluble and -insoluble dietary fibers. We also computed this intake for specific food groups such as cereals, vegetables, and fruits. The mean values obtained from DR and FFQ were compared, and Spearman correlation coefficients between the values obtained from the two methods were computed.

### Cumulative Percent Contribution for Dietary Fiber Intake

The cumulative percent contribution for dietary fiber intake was computed using the DR data.

## RESULTS

Table 1 shows the number of foods used in the computation of dietary fiber intake. A total 456 foods were listed in the developed tables (25% of total number listed in the food composition table). Among the food groups of vegetable origin such as cereals, potatoes and starches, nuts and seeds, pulses, vegetables, fruits, mushroom and seaweeds, the percentage of the food in the food composition tables varied from 39% for potatoes to 63% for mushrooms. We presented the composition table using dietary fiber intake calculations in the Appendix.

**Table 1.** Number of food items used in the computation of dietary fiber

	Basic nutrients	Dietary fiber				
	
	Number of food items in 4th and 5th ed.^1,2^				Newly-developed table	
	
		Number of food items in 4th ed.^1^ (1)	Number of food items in 5th ed.^2^ (2)	Number of food items with substituted composition (3)	Number of food items^3^ (1)+(2)+(3)	Percentage of food items covered^4^
Food group						
Cereals	139	40	5	12	57	41%
Potatoes and starches	36	9	2	3	14	39%
Sugar and sweeteners	25	0	0	2	2	8%
Confectioneries	114	0	0	17	17	15%
Fats and oils	21	0	0	0	0	0%
Nuts and seeds	37	10	2	8	20	54%
Pulses	73	27	12	5	44	60%
Fish and shellfish	389	0	0	12	12	3%
Meats	231	0	0	6	6	3%
Eggs	21	0	0	1	1	5%
Milks	57	0	0	5	5	9%
Vegetables	301	87	46	21	154	51%
Fruits	157	26	24	13	63	40%
Mushrooms	38	13	7	4	24	63%
Seaweeds^5^	46	12	2	4	18	39%
Beverages	73	2	0	2	4	5%
Seasonings and spices	58	1	2	7	10	17%
Processed foods	18	0	0	5	5	28%

Total	1834	227	102	127	456	25%

^1^The Standard Tables of Food Composition in Japan, 4th revised edition by Science and Technology Agency.

^2^Additional food items with dietary fiber contents in the 5th revised edition by Science and Technology Agency.

^3^Total number of food items in the 4th and 5th ed., and food items with substituted dietary fiber composition.

^4^Number of food items in the newly-developed table divided by total number of food items for basic nutrients.

^5^Composition was only available for total dietary fiber.

Tables 2 and 3 show the mean daily intakes of dietary fiber computed from the FFQ and DR, respectively, by area. Because the intakes were remarkably lower in the Ishikawa area compared to those in the other 3 areas, the area difference examined by one-way ANOVA was significant (p<0.05) in both water-soluble and -insoluble fibers.

**Table 2.** Daily intakes of dietary fiber (g/day) assessed with FFQ by area

	Ninohe PHC				Yokote PHC				Saku PHC				Ishikawa PHC				ANOVA p-value
			
	Mean	±	SD	Median	Mean	±	SD	Median	Mean	±	SD	Median	Mean	±	SD	Median	
Men (n=102)	n=24				n=28				n=23				n=27				
Water-soluble	2.7	±	1.5	2.1	2.8	±	1.9	2.3	2.5	±	0.9	2.6	1.4	±	0.9	1.1	0.0015
Water-insoluble	11.6	±	5.0	10.0	11.6	±	7.3	10.0	11.0	±	3.0	10.9	7.0	±	3.4	5.7	0.0024
Total	16.6	±	7.1	14.2	16.7	±	10.0	14.3	15.7	±	4.1	15.4	9.7	±	4.8	7.8	0.0008

Women (n=113)	n=27				n=30				n=28				n=28				
Water-soluble	2.9	±	1.6	2.2	3.1	±	2.4	2.6	3.2	±	2.4	2.5	1.8	±	0.7	1.7	0.0280
Water-insoluble	11.9	±	5.7	10.5	11.9	±	8.0	9.7	13.5	±	10.1	11.0	8.0	±	2.8	7.5	0.0353
Total	16.8	±	7.8	15.2	17.1	±	11.1	14.4	19.0	±	14.0	16.3	11.3	±	4.0	10.4	0.0302

**Table 3.** Daily intakes of dietary fiber (g/day) assessed with DR by area

	Ninohe PHC				Yokote PHC				Saku PHC				Ishikawa PHC				ANOVA p-value
			
	Mean	±	SD	Median	Mean	±	SD	Median	Mean	±	SD	Median	Mean	±	SD	Median	
Men (n=102)	n=24				n=28				n=23				n=27				
Water-soluble	2.5	±	0.8	2.5	2.2	±	0.6	2.1	2.1	±	0.5	2.1	1.7	±	0.6	1.7	0.0005
Water-insoluble	11.8	±	3.0	11.4	10.7	±	2.1	10.6	11.5	±	2.3	11.4	8.8	±	2.3	9.4	0.0001
Total	174	±	3.9	17.2	15.0	±	3.0	14.7	16.2	±	3.2	16.4	12.4	±	3.3	12.7	0.0001

Women (n=113)	n=27				n=30				n=28				n=28				
Water-soluble	2.5	±	0.9	2.2	2.2	±	0.5	2.3	2.2	±	0.6	2.1	1.7	±	0.8	1.5	0.0003
Water insoluble	10.8	±	3.0	9.6	10.5	±	1.9	10.6	10.7	±	2.7	10.7	7.9	±	2.7	7.9	0.0001
Total	16.1	±	4.3	14.8	15.0	±	2.5	15.2	15.4	±	3.7	15.2	11.5	±	3.9	11.8	0.0001

Table 4 shows the mean daily intakes of dietary fiber computed from the DR and FFQ. Total intake assessed with FFQ was 11% higher and 4% lower than those assessed with DR in the analysis of the 4 areas combined (p>0.05). Only water-soluble fiber intake in women was significantly different between DR and FFQ (p<0.01). In area-specific analysis, the mean intake was significantly lower in FFQ than those in DR in Okinawan men (p<0.05). Table 2 also shows the Spearman correlation coefficients between intakes assessed with the two methods. The correlation was slightly higher in crude values (0.48-0.51 in men, 0.40-0.45 in women) than in energy-adjusted values (0.43-0.44 in men, 0.36-0.40 in women).

**Table 4.** Daily intakes of dietary fiber assessed with dietary record and FFQ, and their Spearman correlation coefficients

	Dietary record (g)^1^				FFQ (g)				% difference^2^		Spearman correlation coefficient			
			
	Mean	±	SD	Median	Mean	±	SD	Median		p-value^3^	Crude	Energy-adjusted	Area-adjusted	Energy and area-adjusted
Men (n=102)														
Water-soluble	2.1	±	0.7	2.1	2.3	±	1.5	2.0	10%	0.415	0.48	0.44	0.39	0.44
Water-insoluble	10.6	±	2.7	10.4	10.3	±	5.4	9.5	-4%	0.053	0.51	0.43	0.39	0.42
Total	15.2	±	3.8	14.7	14.6	±	7.5	13.6	-4%	0.060	0.50	0.43	0.36	0.45
Women (n=113)														
Water-soluble	2.1	±	0.7	2.0	2.7	±	2.0	2.2	28%	0.003	0.40	0.36	0.30	0.37
Water-insoluble	10.0	±	2.8	9.6	11.3	±	7.4	9.5	13%	0.458	0.45	0.40	0.35	0.43
Total	14.5	±	4.0	14.4	16.1	±	10.3	14.1	11%	0.797	0.44	0.40	0.33	0.44

^1^Based on two consecutive 7-day records in Ishikawa PHC area and four consecutive 7-day records in the other 3 areas.

^2^(FFQ - DR)/DR (%).

^3^Signed rank test.

Table 5 shows the results of the same analysis for dietary fiber derived from cereals, vegetables, and fruits. Mean intake of vegetables assessed with FFQ was significantly lower than that assessed with DR (p<0.001), whereas mean intake of fruits assessed with FFQ was significantly higher than that assessed with DR (p<0.001). The correlation coefficients were high for cereals in men, and moderate in women (0.60 and 0.36 for crude value, and 0.49 and 0.17 for energy-adjusted values, respectively). The correlation coefficients were low for vegetables in both men and women (0.26 and 0.27 for crude value, and 0.18 and 0.15 for energy-adjusted values, respectively). The correlation coefficients were high for fruits in both men and women (0.62 and 0.49 for crude value, and 0.46 and 0.22 for energy-adjusted values, respectively).

**Table 5.** Daily intakes of dietary fiber by main food source assessed with DR and FFQ, and their Spearman correlation coefficients

Sex	DR(g)^1^				FFQ (g)				% difference^2^		Crude	Energy- djusted^4^
			
Food group	Mean	±	SD	Median	Mean	±	SD	Median		p-value^3^		
Men (n=102)												
Cereals	3.0	±	0.8	2.9	2.7	±	0.9	2.7	-10%	<0.001	0.60	0.49
Vegetables	5.8	±	1.8	5.7	3.9	±	2.7	3.3	-34%	<0.001	0.26	0.18
Fruits	1.4	±	1.1	1.2	2.8	±	3.0	2.1	98%	<0.001	0.62	0.46
Other^5^	4.9	±	1.6	4.8	5.2	±	2.9	5.0	7%	0.489	0.49	0.36
Women (n=113)												
Cereals	2.3	±	0.5	2.3	2.5	±	0.8	2.3	8%	0.111	0.36	0.17
Vegetables	5.6	±	1.9	5.3	4.6	±	3.6	3.9	-18%	<0.001	0.27	0.15
Fruits	1.9	±	1.2	1.7	3.6	±	3.8	2.5	90%	<0.001	0.49	0.22
Other^5^	4.7	±	1.6	4.7	5.4	±	3.8	4.8	14%	0.224	0.37	0.35

^1^Based on two consecutive 7-day records in Ishikawa PHC area and four consecutive 7-day records in the other 3 areas.

^2^(FFQ - DR)/DR (%).

^3^Signed rank test.

^4^Energy was adjusted by residual method.

^5^Other: includes dietary fiber from all foods except for cereals, vegetables and fruits.

Table 6 shows the mean daily intakes of dietary fiber obtained by DR within the quintile of intake estimated by FFQ. The intakes by DR increased steadily according to the increase in the intake category determined by FFQ except in the fourth quintile in men. The mean intakes of the highest quintiles were 1.42-1.68 times higher than those of the lowest quintiles.

**Table 6.** Mean intake of dietary fiber from DR^1^ within quintile of intake determined by FFQ^2^

	Quintile of dietary fiber intake according to FFQ																		

	Lowest			Second				Third				Fourth				Highest			
				
	Mean	±	SDn	Mean	±	SD	ratio^3^	Mean	±	SD	ratio^3^	Mean	±	SD	ratio^3^	Mean	±	SD	ratio^3^
Men	n=20			n=21				n=20				n=21				n=20			
Water-soluble	1.6	±	0.7	2.0	±	0.5	1.29	2.3	±	0.7	1.43	2.1	±	0.6	1.35	2.6	±	0.6	1.68
Water-insoluble	8.2	±	2.4	10.5	±	2.0	1.29	10.9	±	2.5	1.34	10.4	±	1.8	1.28	13.1	±	2.4	1.61
Total	11.6	±	3.4	15.2	±	2.5	1.31	15.4	±	4.1	1.32	15.1	±	2.5	1.30	18.5	±	3.2	1.59

Women	n=22			n=23				n=23				n=23				n=22			
Water-soluble	1.6	±	0.6	2.1	±	0.7	1.25	2.2	±	0.7	1.32	2.3	±	0.9	1.41	2.5	±	0.6	1.51
Water-insoluble	7.9	±	2.2	9.1	±	2.2	1.16	10.5	±	2.5	1.34	10.8	±	3.2	1.37	11.6	±	2.5	1.47
Total	11.5	±	3.5	14.0	±	3.4	1.22	14.8	±	2.9	1.29	15.8	±	4.8	1.38	16.3	±	3.4	1.42

^1^DR, dietary records.

^2^FFQ, food frequency questionnaire.

^3^Ratio compared to the lowest quintile.

Table 7 compares the FFQ with DR for dietary fiber based on joint classification by quintile (%). The 33-40% and 27-28% of subjects were included in the same category in men and women, respectively. Only 0-2% of subjects were included in the extreme categories.

**Table 7.** Comparison of FFQ^1^ with DR^2^ for dietary fiber based on joint classification by quintile (%)

	Men (n=102)			Women (n=113)		
	
	Same category	Adjacent category	Extreme category	Same category	Adjacent category	Extreme category
Water-soluble	33	34	2	27	36	1
Water-insoluble	40	31	1	27	39	0
Total	34	38	2	28	35	2

^1^FFQ, food frequency questionnaire.

^2^DR, dietary records.

Tables 8-10 show the cumulative percent contribution of the top 20 foods for water-soluble, -insoluble, and total dietary fiber intakes, respectively. Some vegetables which were ranked within the top 20 foods, (e.g., edible burdock, eggplant) were not included in the FFQ.

**Table 8.** Cumulative % contribution of the top 20 foods for water soluble dietary fiber assessed by DR

Food code^1^	Description^1^	g/day	Cumulative^2^ percent
Men (n=102)			
7-29	Natto, fermented soybeans/Itohiki-natto	0.16	7.4
12-31a	Edible burdock/Root/Raw	0.15	14.3
12-56a	Daikon, Japanese radish/Root/Raw	0.13	20.4
7-32c	Miso/Rice-koji Miso/Dark yellow type	0.12	25.9
12-94a	Carrot/Root/Raw	0.11	30.8
13-17b	Satsuma mandarins	0.10	35.4
12-117a	Spinach/Leaves/Raw	0.09	39.7
13-88	Apples/Raw fruit	0.09	43.7
1-10a	Wheat flour/Soft flour/First grade	0.07	47.0
1-26a	Chinese noodle i.e., Lao Mien/Raw, dry form	0.06	49.7
12-8a	Green soybeans/Beans, immature/Raw	0.05	52.2
12-95	Garlic/Bulb	0.05	54.6
12-14a	Okura/Pods, immature/Raw	0.04	56.6
12-18a	Pumpkin and squash/Squash/Raw	0.04	58.7
12-58	Daikon, Japanese radish/Takuan-zuke	0.04	60.4
1-13a	Wheat (Breads)/White bread/On the market	0.04	62.1
12-114a	Broccoli/Head/Raw	0.04	63.8
1-24b	Japanese noodles/Somen Hiyamugi/Boiled	0.03	65.4
7-21a	Tofu and Abura-age/Tofu, soybean curd	0.03	67.0
12-25a	Cucumber/Fruit/Raw	0.03	68.4
Women (n=113)			
7-29	Natto, fermented soybeans/Itohiki-natto	0.14	6.7
12-31a	Edible burdock/Root/Raw	0.14	13.1
13-17b	Satsuma mandarins	0.14	19.4
12-56a	Daikon, Japanese radish/Root/Raw	0.11	24.8
13-88	Apples/Raw fruit	0.11	29.7
7-32c	Miso/Rice-koji Miso/Dark yellow type	0.10	34.4
12-94a	Carrot/Root/Raw	0.10	39.0
12-117a	Spinach/Leaves/Raw	0.09	43.3
1-10a	Wheat flour/Soft flour/First grade	0.06	46.3
12-18a	Pumpkin and squash/Squash/Raw	0.05	48.9
1-26a	Chinese noodle i.e., Lao Mien/Raw, dry form	0.05	51.1
1-13a	Wheat (Breads)/White bread/On the market	0.04	53.1
12-14a	Okura/Pods, immature/Raw	0.04	55.1
12-114a	Broccoli/Head/Raw	0.04	57.0
13-27	Kaki, Japanese Dried fruit	0.04	58.8
12-8a	Green soybeans/Beans, immature/Raw	0.04	60.6
12-95	Garlic/Bulb	0.04	62.3
2-5a	Sweet potatoes/Tuber/Raw	0.03	63.9
12-58	Daikon, Japanese radish/Takuan-zuke	0.03	65.3
12-25a	Cucumber/Fruit/Raw	0.03	66.7

^1^Food codes and descriptions correspond to those of the Standard Tables of Food Composition, 4th revised edition in Japan by Science and Technology Agency.

^2^Data on subjects in Ishikawa PHC (14 days data) were counted twice for 28-day data.

**Table 9.** Cumulative % contribution of the top 20 foods for water-insoluble dietary fiber assessed by DR

Food code^1^	Description^1^	g/day	Cumulative^2^ percent
Men (n=102)			
1-41d	Rice (Paddy rice)/Grains/Well-milled rice	1.83	17.3
7-32c	Miso/Rice-koji Miso/Dark yellow type	0.68	23.7
12-24a	Cabbage/Head/Raw	0.49	28.3
12-94a	Carrot/Root/Raw	0.40	32.0
14-7a	Shiitake/Dried/Uncooked	0.37	35.5
12-117a	Spinach/Leaves/Raw	0.31	38.4
7-29	Natto, fermented soybeans/Itohiki-natto	0.30	41.2
13-88	Apples/Raw fruit	0.29	43.9
2-11a	Potatoes/Tuber/Raw	0.27	46.5
12-56a	Daikon, Japanese radish/Root/Raw	0.26	48.9
12-70a	Onion/Bulb/Raw	0.24	51.2
12-87a	Eggplant/Fruit/Raw	0.21	53.2
12-18a	Pumpkin and squash/Squash/Raw	0.20	55.1
12-96	Welsh onion/Leaf and leafsheath, blanched	0.20	56.9
1-13a	Wheat (Breads)/White bread/On the market	0.17	58.5
12-31a	Edible burdock/Root/Raw	0.16	60.1
13-64	Bananas/Raw fruit	0.15	61.5
2-3	Konnyaku: block type/Made with fine powder	0.14	62.8
13-26a	Japanese Persimmons/Raw fruit/Hard type	0.14	64.1
12-114a	Broccoli/Head/Raw	0.14	65.4
Women (n=113)			
1-41d	Rice (Paddy rice)/Grains/Well-milled rice	1.18	11.8
7-32c	Miso/Rice-koji Miso/Dark yellow type	0.58	17.6
12-24a	Cabbage/Head/Raw	0.43	21.9
12-94a	Carrot/Root/Raw	0.37	25.6
13-88	Apples/Raw fruit	0.36	29.2
14-7a	Shiitake/Dried/Uncooked	0.34	32.6
12-117a	Spinach/Leaves/Raw	0.31	35.8
7-29	Natto, fermented soybeans/Itohiki-natto	0.28	38.5
12-18a	Pumpkin and squash/Squash/Raw	0.25	41.1
2-11a	Potatoes/Tuber/Raw	0.25	43.6
12-70a	Onion/Bulb/Raw	0.23	45.9
12-56a	Daikon, Japanese radish/Root/Raw	0.23	48.2
1-13a	Wheat (Breads)/White bread/On the market	0.21	50.3
12-87a	Eggplant/Fruit/Raw	0.19	52.2
13-17b	Satsuma mandarins	0.19	54.1
13-26a	Japanese Persimrnons/Raw fruit/Hard type	0.18	55.8
12-96	Welsh onion/Leaf and leafsheath, blanched	0.17	57.6
13-64	Bananas/Raw fruit	0.16	59.2
12-31a	Edible burdock/Root/Raw	0.15	60.7
12-114a	Broccoli/Head/Raw	0.15	62.2

^1^Food codes and descriptions correspond to those of the Standard Tables of Food Composition, 4th revised edition in Japan by Science and Technology Agency.

^2^Data on subjects in Ishikawa PHC (14-day data) were counted twice for 28-day data.

**Table 10.** Cumulative % contribution of the top 20 foods for total dietary fiber assessed by DR

Food code^1^	Description^1^	g/day	Cumulative^2^ percent
Men (n=102)			
1-41d	Rice (Paddy rice)/Grains/Well-milled rice	1.83	12.1
7-32c	Miso/Rice-koji Miso/Dark yellow type	0.80	17.4
12-24a	Cabbage/Head/Raw	0.51	20.8
12-94a	Carrot/Root/Raw	0.51	24.1
7-29	Natto, fermented soybeans/Itohiki-natto	0.46	27.1
12-117a	Spinach/Leaves/Raw	0.40	29.8
12-56a	Daikon, Japanese radish/Root/Raw	0.39	32.4
14-7a	Shiitake/Dried/Uncooked	0.38	34.9
13-88	Apples/Raw fruit	0.37	37.4
15-35a	Wakame/Dried/Dried	0.31	39.4
12-31a	Edible burdock/Root/Raw	0.31	41.5
2-11a	Potatoes/Tuber/Raw	0.30	43.4
12-70a	Onion/Bulb/Raw	0.26	45.2
12-18a	Pumpkin and squash/Squash/Raw	0.24	46.8
13-17b	Satsuma mandarins	0.23	48.3
12-87a	Eggplant/Fruit/Raw	0.23	49.8
1-13a	Wheat (Breads)/White bread/On the market	0.21	51.2
11-2	Liquid milk/Ordinary liquid milk	0.21	52.5
12-96	Welsh onion/Leaf and leafsheath, blanched	0.21	53.9
1-26a	Chinese noodle i.e, Lao Mien/Raw, dry form	0.18	55.0
Women (n=113)			
1-41d	Rice (Paddy rice)/Grains/Well-milled rice	1.18	8.1
7-32c	Miso/Rice-koji Miso/Dark yellow type	0.68	12.8
12-94a	Carrot/Root/Raw	0.47	16.1
13-88	Apples/Raw fruit	0.46	19.2
12-24a	Cabbage/Head/Raw	0.45	22.4
7-29	Natto, fermented soybeans/Itohiki-natto	0.42	25.3
12-117a	Spinach/Leaves/Raw	0.41	28.1
14-7a	Shiitake/Dried/Uncooked	0.36	30.6
12-56a	Daikon, Japanese radish/Root/Raw	0.34	32.9
15-35a	Wakame/Dried/Dried	0.33	35.2
13-17b	Satsuma mandarins	0.32	37.4
12-18a	Pumpkin and squash/Squash/Raw	0.31	39.6
12-31a	Edible burdock/Root/Raw	0.29	41.6
2-11a	Potatoes/Tuber/Raw	0.27	43.4
1-13a	Wheat (Breads)/White bread/On the market	0.26	45.2
12-70a	Onion/Bulb/Raw	0.25	46.9
11-2	Liquid milk/Ordinary liquid milk	0.24	48.6
13-26a	Japanese Persimmons/Raw fruit/Hard type	0.20	50.0
12-87a	Eggplant/Fruit/Raw	0.20	51.4
12-114a	Broccoli/Head/Raw	0.19	52.7

^1^Food codes and descriptions correspond to those of the Standard Tables of Food Composition, 4th revised edition in Japan by Science and Technology Agency.

^2^Data on subjects in Ishikawa PHC (14-day data) were counted twice for 28-day data.

## DISCUSSION

The mean intake of dietary fiber assessed with FFQ was higher in fruits and lower in vegetables than those assessed with DR. This result indicated a possible problem of standard portion size used in FFQ for vegetables and/or fruits. The observed vegetable and fruit fiber intakes were 5.8 and 1.4 g/day in men and 5.6 and 1.9 g/day in women, respectively (Table 5). The ratio of vegetable to fruit fiber, 4.2 in men and 2.9 in women, was higher than the 2.3 previously reported,^5^ and comparable with or slightly lower than the 4.1 in men and 3.3 in women in a different study.^6^

The correlation coefficients observed in the present study (i.e., 0.36-0.51 in men and 0.30-0.45 in women) were slightly lower than those in questionnaires previously developed in Japan (i.e., 0.39-0.61 and 0.63-0.68).^11^^,^^12^ These correlation coefficients dropped slightly when energy was adjusted for, and then dropped even further when area was adjusted for. However, when both energy and area were adjusted for, the correlation coefficients improved compared to the values with only area adjustment. When the possible association of intake between FFQ and DR was examined by the main food source of dietary fiber, the correlation coefficients were high or moderate for dietary fiber from cereals and fruits, but were low for dietary fiber from vegetables.

One of the limitations of our study is that the intakes of dietary fiber obtained from DR might not be suitable as the gold standard due to the incompleteness of the food composition table. We developed the food composition table not only for the FFQ but also for DR, which might have induced overestimation of the validity of the FFQ. Possible overestimation of its validity could not be examined because the biomarkers for dietary fiber were not available. The Standard Tables of Food Composition in Japan, 5th revised edition with dietary fiber composition for all foods,^8^ was published after this food composition table was developed. The validity of the FFQ for dietary fiber should be reexamined using this new table.

Foods listed among the top 20 contributors to dietary fiber intake were somewhat different from the foods important for estimates of other nutrient intake; edible burdock, garlic, eggplant and konnyaku were not listed as important contributors of any other nutrients.^13^^-^^15^ The lack of these foods in FFQ might have lowered the validity of FFQ for ranking individuals by dietary fiber intake. In addition, the limited number of vegetables contributing to dietary fiber intake in FFQ might have been due to the significantly lower estimates of mean intakes by the FFQ than by the DRs.

In conclusion, the validity of the FFQ used for the 5-year follow-up survey in JPHC Study to estimate dietary fiber intake was moderately high. The FFQ can be used for ranking individuals for dietary fiber intakes in epidemiologic studies, despite the difficulty in estimating mean intake.
